# An early microvascular training program of dental intern students and junior residents: a comparative prospective study

**DOI:** 10.1186/s13005-023-00360-7

**Published:** 2023-05-16

**Authors:** Sadam Ahmed Elayah, Xiang Liang, Karim Ahmed Sakran, Wael Telha, Maged Ali Al-Aroomi, Hamza Younis, Sarah A. Alqurmoti, Omar Ghaleb, Hao Cui, Weiqi Wang, Sijia Na

**Affiliations:** 1 Key Laboratory of Shaanxi Province for Craniofacial Precision Medicine Research, Department of Oral and Maxillofacial Surgery, College of Stomatology, Xi’an Jiaotong University, Xi’an, Shaanxi China; 2 State Key Laboratory of Oral Diseases and National Clinical Research Centre for Oral Diseases, Department of Oral and Maxillofacial Surgery, West China Hospital of Stomatology, Sichuan University, Chengdu, China; 3 Department of Oral and Maxillofacial Surgery, Ibb University, Ibb, Yemen; 4 State Key Laboratory of Military Stomatology, National Clinical Research Center for Oral Disease, Department of Oral and Maxillofacial Surgery, School of Stomatology, Air Force Military Medical University, Xi’an, Shaanxi China

**Keywords:** Intern students, Junior residents, Microsurgical technique, Microsurgical instruments, Anastomosis

## Abstract

**Background:**

Clinical instructional strategies and the climate in which teaching and learning take place have a significant impact on the quality of dental education. Therefore, this study aimed to evaluate the impact of early microsurgery training on the skills of dental intern students who are planning to join an oral and maxillofacial surgical field (DIS) as compared with junior residents within an oral and maxillofacial surgery department who had no microsurgery experience (JR).

**Methods:**

A total of 100 trainees, 70 were DIS, while the other 30 were JR. The average age was 23.87 ± 2.05 years for DIS group and 31.05 ± 3.06 for JR group. All trainees attended a microsurgical course (theoretical and practical parts) for seven days within a Microvascular Laboratory for Research and Education of a university-affiliated tertiary hospital. Two blinded examiners had assessed the performance of trainees independently using a specific scoring system. The independent sample t-test was used to compare the effect of microsurgery training between DIS and JR groups. The significance level was set at 0.05.

**Results:**

The DIS group had showed higher attendance rate than JR group (p < 0.01), with a lower absence score in DIS than JR groups (0.33 ± 0.58 vs. 2.47 ± 1.36). The total score of the theoretical test was significantly different between both groups (p < 0.01). In this context, the DIS group had revealed higher total score than JR group (15.06 ± 1.92 vs. 12.73 ± 2.49). In term of tissue preservation, there was a significant difference between both groups, with the DIS had better performance score than JR (1.49 ± 0.51 vs. 0.93 ± 0.59). Further, the practical exam score was significantly higher in DIS group than JR group (p < 0.01).

**Conclusion:**

Overall, the performance of dental intern students was favourably compared with junior residents in most aspects. Therefore, it is promising and essential for dental colleges to add a microsurgery course to the curriculum of dental intern students who plan to specialize in oral and maxillofacial surgery.

## Background

Clinical instructional strategies and the climate in which teaching and learning take place have a significant impact on the quality of dental education. Therefore, attempts to improve clinical teaching should focus on both the instructor and the educational environment [1–3]. As a dental educator, one of your main responsibilities is to ensure that new graduates have the necessary skills and character attributes to begin their employment. It is theorized that the effectiveness of undergraduate training impacts students’ performance and their ability to complete tasks to a significant level [4]. Thus, the dentistry school curriculum is continuously modified to fit advancements in current dental practice, the demands on the new dentists, and the developing structure of the dental profession [5].

    One of the essential clinical innovations of the past decades is microsurgery [6, 7]. Microsurgical techniques are becoming more necessary for various surgical interventions. The process of gaining experience in microsurgery seems to differ widely amongst various specializations, partially due to the frequency of microsurgical approaches [8]. Microsurgical learning is a challenging process that needs a considerable amount of time, exceptional manual expertise, and patience, in addition to continual practicing and emotional balance [9–12]. Typically, classical microsurgery training programs usually begin after junior residents’ shifts. During this time, they are exposed to social pressure, high-intensity clinical tasks, and insufficient training and learning opportunities, while undergraduate internships lack surgical clinical skills, and the effect of microsurgery training is not clear.

    Medical students often feel inadequate for the obligations of a surgical internship because of a lack of exposure to resident tasks before beginning residency [13]. Early microsurgical training for young doctors may encourage them to practice and learn principles early in their clinical studies [14]. Typically, an intensive individual experiment is a starting point for acquiring experience in microsurgery [10]. Early undergraduate training could accelerate learning by enhancing excellent surgical skills and “habits” and avoiding self-taught training [8]. In this context, most relevant studies focused on medical students (undergraduate and internship students) [8, 12–17]. Meanwhile, they neglected the dental internship students who plan to enter a surgical field and junior residents within an oral and maxillofacial surgery department. Therefore, the design of microsurgery training content and timing become controversial among oral and maxillofacial surgeons and educators as well. Thus, this study aimed to evaluate and compare the impact of early microsurgery training on the skills of dental intern students who plan to join an oral and maxillofacial surgical field with junior residents within an oral and maxillofacial surgery department, who had no microsurgery experience.

## Materials and methods

### Trainees

A total of 100 trainees, 70 were dental intern students who are planning to join an oral and maxillofacial surgical field (DIS), while the other 30 were junior residents within an oral and maxillofacial surgery department who had no microsurgical experience (JR). The average age was 23.87 ± 2.05 years for DIS group and 31.05 ± 3.06 for JR group. Based on Helsinki principles, all trainees attended a microsurgical course (theoretical and practical parts) for seven days within a Microvascular Laboratory for Research and Education of a university-affiliated tertiary hospital. The trainees were randomly divided into 25 groups, with 4 trainees in each group. The trainee’s size was conducted based on previous comparable studies [8, 18, 19]. Based on ARRIVE guidelines, all rat experiments were carried out at the Advanced Science Research Center, Department of Animal Resources, with approval from Xian Jiaotong University, Animal Research Committee (ID: 2022 − 1522), and has been written informed consents were obtained from all trainees and instructors (two experienced microsurgeons) who followed the Helsinki principles. For the duration of this training course, all trainees were released from daily routine work.

### Microsurgical equipment

Keoda ASOM-4B microscopes (China) were given extra tubes to observe and instruct the trainees during the microsurgical workouts.

### Microsurgical training program

The training course is divided into two sections: theoretical and practical, with a particular emphasis on the practical section (Table 1). Each theoretical session was for 50 min [8], during which educators instructed the trainees on fundamental microsurgical approaches. This included details on handling the micro-instruments, suturing techniques for various minor vascular anastomosis, and their medical applications. Lectures on sterilization and cleaning micro-instruments were presented. Each practical session lasted for 120 min, and the program’s practical section consisted of a step-by-step training schedule using models and rats that have been previously reported and commonly used in practical training courses [9, 18, 20, 21]. Every trainee received equal opportunities from each instructional content session.

**Table 1:** Microvascular training program

Day	Theoretical training part (50 min)	Practical training part (120 min)
1	Basic introduction of the microsurgical technique [9, 22]	The latex model with 10 − 0 suturing [23] &Training with gauze for practicing suture handling [24]
2	Cleaning and storage of micro-instruments.	Preparation of rat femoral artery and vein, rat tail artery under a microscope
3	Basic microvascular surgical skills [11]	End-to-end anastomosis of rat femoral artery (interrupted suturing) with 10 − 0 sutures.
4	Application of microsurgical techniques [25–27].	End-to-end anastomosis of rat femoral vein (Continuous suturing) with 10 − 0 suturing.
5	Monitoring of free flaps [28–30]	Preparation of rat inguinal flap
6	Risk factors for microsurgery failure [31, 32]	Preparation and transplantation of rat inguinal flaps.
7	Theoretical Exam	Practical Exam

### Assessment of knowledge and skills

At the 7th day of the course, both practical and theoretical examinations were conducted, and the knowledge and skills acquired during this training course were evaluated using an objective grading system [8, 33]. The theoretical assessment consisted of twenty multiple-choice questions regarding basic knowledge in microsurgery, which were discussed in this course.

**Fig. 1:**
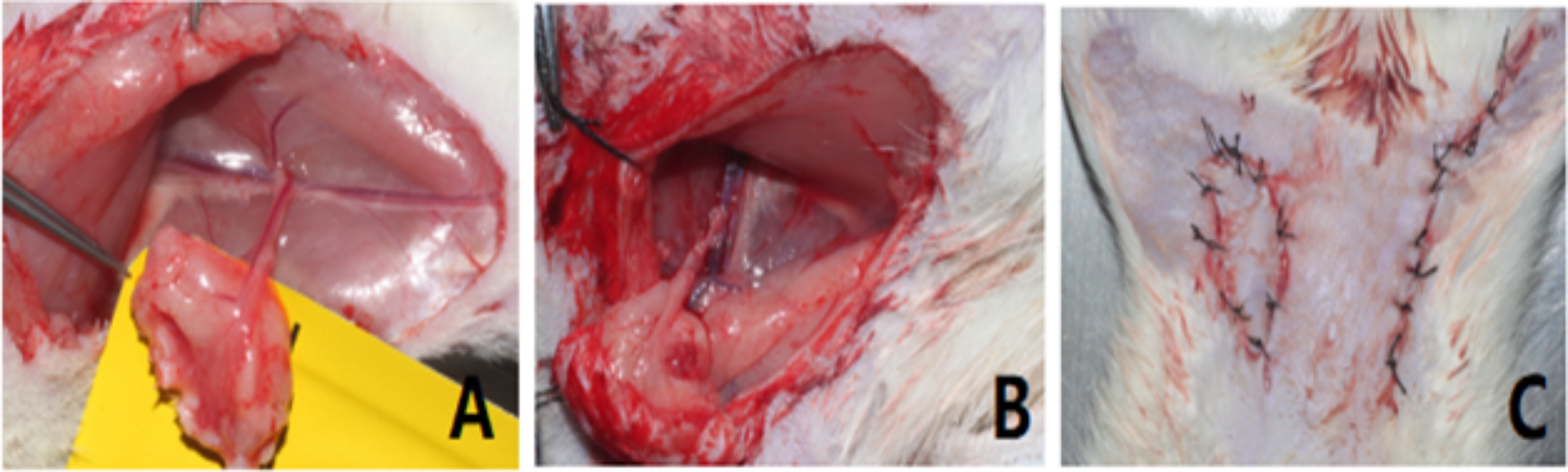
**A**: Preparation of rat inguinal flap; **B**. End-to-end anastomosis of femoral artery and vein; **C**. Flap transplantation

In the practical part, the trainees had to independently carry out end-to-end anastomosis of one another rat femoral vein and artery (Fig. 1).

While the trainees were directly instructed during the training by two experienced microsurgeons, the examinations were blindly assessed and scored by two other blinded microsurgeons, who had to judge the students separately and independently of each other to achieve greater reliability. A scoring system was used to assess the performance of trainees on the vascular anastomosis, which ranged from − 2 to + 2 scores, evaluated according to predefined objective criteria such as procedure time, handling of micro-instruments, proper needle handling, safe knot technique, proper thread cutting, distance of suture from vessel margins, proper vessel preparation, and tissue protection technique (Table 2).

**Table 2:** Evaluation criteria for the post-training microvascular program

	Evaluation indicators	Index evaluation method
1	Operation time	Time from flap preparation to completion of the femoral vein and artery end-to-end anastomosis
2	Proper dealing with Microscope	Correct use of microsurgical instruments: 2 points Device usage error: -2 points
3	Proper use of needles	Minor deformation of the suture needle: 2 points Large deformation of the suture needle: -2 points
4	suture knotting technique	No anastomotic stenosis: 2 points Suture the anterior and posterior walls of the blood vessel: -2 points
5	Proper cutting of the thread	The length of the thread is appropriate and uniform: 2 points The length of the remaining thread is inappropriate and uneven: -2 points
6	Distance of the suture of vessel’s margins	The needle distance and margin are correct and uniform, and the blood vessel is well valgus: 2 points; Leakage or complete inversion of blood vessels: -2 points
7	Proper preparation of blood vessels	The blood vessels and surrounding tissues of the operation segment are entirely separated, and the length of the adventitial stripping around the anastomosis is correct: 2 points The blood vessels and surrounding tissues of the operation segment are not separated, and the adventitia around the anastomosis is not peeled off: -2 points.
8	Tissue protection technology	The surgical field is clear without damage to surrounding normal tissues: 2 points. More bleeding in the surgical field, serious damage to surrounding tissues, and death of Sprague-Dawley (SD) rats: -2 points

### Data analysis

SPSS v. 19.0 statistical software was used to conduct the statistical analysis of this study. Descriptive statistics are presented as the mean and standard deviation. The impact of microsurgery training between DIS and JR groups was compared using the independent sample t-test. The significance level was set at 0.05.

## Results

A total of 100 trainees, 70 were dental intern students who are planning to join an oral and maxillofacial surgical field (DIS), while the other 30 were junior residents within an oral and maxillofacial surgery department who had no microsurgical experience (JR). The average age was 23.87 ± 2.05 years for DIS group and 31.05 ± 3.06 for JR group. Regarding the attendance rate of trainees, our findings showed a highly significant difference between both groups (p < 0.01), and the score of absence time for DIS was less than that of JR (0.33 ± 0.58 vs2.47 ± 1.36).

For theoretical section, a significant difference was reported between both groups (p < 0.01), and the scores of DIS were higher than those of JR (15.06 ± 1.92 vs12.73 ± 2.49).

In term of practical section, the criterion for tissue preservation showed a significant difference, with DIS group had better performance than JR group (1.49 ± 0.51 vs. 0.93 ± 0.59) (Table 3). The other objective criteria had shown non-significant higher scores in DIS group compared with JR group. On the other hand, the results of the practice exam showed a statistically significant difference between DIS and JR (p < 0.01), and the scores of DIS were higher than those of JR (Table 3).

**Table 3:** ; Assessment of DIS and JR results after the examination

Index		Score (M ± S)		*p-value*
		DIS	JR	
**Number of days absent**		0.33 ± 0.58	2.47 ± 1.36	0.01
**Theoretical part (20 points)**		15.06 ± 1.92	12.73 ± 2.49	0.001
	Operation time	1.37 ± 0.59	1.27 ± 0.59	0.572
	Proper dealing with Microscope	1.40 ± 0.65	1.33 ± 0.49	0.724
	Proper handling of microsurgical needles	1.40 ± 0.55	1.33 ± 0.49	0.688
**Practical part**	Safe knotting technique	1.54 ± 0.51	1.13 ± 0.74	0.280
	Proper cutting of the thread	1.57 ± 0.50	1.20 ± 1.56	0.250
	Distance of the suture of the vessel’s margins	1.49 ± 0.56	1.47 ± 0.52	0.911
	Proper preparation of vessels	1.43 ± 0.50	1.20 ± 0.41	0.128
	Tissue preserving technique	1.49 ± 0.51	0.93 ± 0.59	0.01
Total score		11.69 ± 1.58	9.87 ± 1.55	0.01

**DIS;** dental intern students who are planning to join an oral and maxillofacial surgical field. **JR;** junior residents within an oral and maxillofacial surgery department who had no microsurgical experience.

## Discussion

Since the world’s first severed hand reimplantation in 1963 was accomplished by Chen Zhongwei, the application of China’s microsurgery has progressed in orthopedics, burns, plastic surgery, and other surgical fields [34]. However, the microsurgery training in the field of oral and maxillofacial surgery still lacks systematic and scientific Instructional design, especially in developing countries. The present study amid to evaluate and compare the effect of early microsurgery training on the skills of dental intern students who plan to join an oral and maxillofacial surgical field with junior residents within an oral and maxillofacial surgery department who had no microsurgery experience. According to our knowledge, this is the first prospective study comparing the DIS and JR in term of microsurgical training.

According to the current findings, DIS presented greater enthusiasm, interest, and their attendance rate was significantly higher than that of JR. Further, DIS performed better on the theoretical test than did JR, demonstrating that DIS had a higher ability for theoretical learning and could more effectively assimilate new knowledge. These outcomes were consistent with Mücke et al. [8], who reported that the primary cause of medical residents’ absence was additional work interference, followed by interpersonal obligations. Similarly, Lascar et al. [11], Brown et al. [33], Mücke et al. [30], and Kelly et al. [35]concluded that there is little opportunity to learn microsurgery techniques during the residency program due to the demanding workload as well as restriction from devoting enough time to microsurgery training.

Contrary to our expectations, a significant difference in tissue preservation was noticed during the trainees’ skills’ blinded evaluation at the end of this course (DIS better than JR), which may showcase the significance of early microsurgical training in medical school. On the other hand, the overall scores for the practical part were higher in DIS than JR. Lindeman et al. [36] reported that early microsurgical training programs give senior medical students who want to specialize in surgery a chance to be more concerned about in-patient care. Cataldo et al. [37] reported that even if the students do not wish to specialize in microsurgery, an early microsurgery program has a beneficial impact on their ability to perform macroscopic surgery such as tissue handling and manipulation, becoming familiar with surgical instruments, performing the proper dissection of soft tissue, and obtaining more control over the placement of sutures. Our findings concluded that due to the surgical operation skills developed by the residents in the clinic for many years, they did not complete the practice exam in full accordance with the instructor’s requirements. Mücke et al.[8] reported that students with no previous experience with macroscopic surgery could learn quickly using model-based courses. On the other hand, it may be possible to minimize the potential for undesirable practices to be carried over from macroscopic to microscopic surgery by beginning microsurgical courses without any or little surgical experience [8]. Our conclusion was consistent with Klingensmith et al. [17], who concluded that the best way to promote skill progression during surgical residency is to build a strong base of skills early during the internship stage. The anastomoses are mainly depending on how the tissue is handled. A Failure in microsurgery may result in serious side effects such as flap necrosis, ischemia, or permanent failure.

The rate of learning in microsurgery is slow and greatly influenced by the quantity and quality of microsurgical training [38]. Based on the days absent rate (0.33 ± 0.58 vs. 2.47 ± 1.36), all trainees showed a strong desire to take maximum possible benefit from the training opportunity program. At the end of this microsurgery course, about 93% of the trainees believed the microsurgical training course was absolutely necessary. Thus, practical training courses become necessary for medical students, junior residents, and it may be helpful to practice their fundamental set of microsurgical skills early in their residency training program [15, 33, 35].

The current study still needs to be further adjusted and improved in the program content and the time required for this course. Thus, these limitations should be considered in further studies.

## Conclusion

The main conclusions of this comparative study are as follows:

1. Dental intern students have higher attendance score owing to their time management flexibility, which allow them to attend microsurgical classes without feeling pressured or carrying an extra workload.
2. Dental intern students’ performance was higher on both portions of the test, highlighting their strong desire to acquire new knowledge and clinical approaches.
3. Early microsurgical training programs provide dental intern students who plan to specialize in oral and maxillofacial surgery the chance to become more concerned with clinical outcomes and get them ready for the subsequent level of responsibility.

Therefore, this study recommends that it is worthy and essential for dental colleges to add a microsurgery course to the curriculum of dental intern students who plan to specialize in oral and maxillofacial surgery.

## Data Availability

The datasets used and/or analysed during the study are available from the corresponding author on reasonable request.

## List of abbreviations

DIS: dental intern students who are planning to join an oral and maxillofacial surgical field.
JR: junior residents within an oral and maxillofacial surgery department who had no microsurgery experience.
